# Papillary renal neoplasm with reverse polarity: A clinicopathological and molecular genetic characterization of 16 cases with expanding the morphologic spectrum and further support for a novel entity

**DOI:** 10.3389/fonc.2022.930296

**Published:** 2022-07-22

**Authors:** Miaomiao Shen, Xiaona Yin, Yanfeng Bai, Huizhi Zhang, Guoqing Ru, Xianglei He, Xiaodong Teng, Guorong Chen, Ming Zhao

**Affiliations:** ^1^ Cancer Center, Department of Pathology, Zhejiang Provincial People’s Hospital, Affiliated People’s Hospital, Hangzhou Medical College, Hangzhou, China; ^2^ Department of Pathology, Hangzhou Women’s Hospital, Hangzhou, China; ^3^ Department of Pathology, The First Affiliated Hospital, Zhejiang University School of Medicine, Hangzhou, China; ^4^ Department of Pathology, Ningbo Diagnostic Pathology Center, Ningbo, China; ^5^ Department of Pathology, The First Affiliated Hospital, Wenzhou Medical University, Wenzhou, China

**Keywords:** papillary renal neoplasm with reverse polarity, papillary renal cell carcinoma, GATA3, *KRAS* mutation, next-generation sequencing

## Abstract

Papillary renal neoplasm with reverse polarity (PRNRP) is a recently described, rare renal tumor that differs clinically, morphologically, and molecularly from papillary renal cell carcinoma (RCC). To further characterize the pathological spectrum of this rare tumor, in this study, we retrospectively identified 16 cases of PRNRP from three institutions to comprehensively investigate the clinicopathological and molecular genetic features, using immunohistochemistry (IHC), fluorescence *in-situ* hybridization (FISH), and targeted next-generation sequencing (NGS). The patients included nine men and seven women, with age ranging from 47 to 80 years (median = 67.5 years, mean = 65 years). The tumor size ranged from 0.4 to 9.5 cm in the greatest dimension (median = 1.8 cm, mean = 2.6 cm). Most tumors (12/16) were incidentally identified by imaging studies. By AJCC stage, 15 were categorized as pT1 and 1 was pT2. Follow-up showed no recurrences, metastases, or disease-related deaths in all the 16 patients. Grossly, 14 cases demonstrated at least a partially cystic appearance. Microscopically, all PRNRPs except 1 (case 13) were composed predominantly of thin, branching papillary architecture covered by a single layer of cuboidal cells with finely granular cytoplasm, and low-grade nuclei typically located toward the apical surface away from the basement. Case 13 consisted mostly of solid, densely packed tubules with only a minor papillary component (5%). Other commonly seen histological features included hyalinized or edematous papillae (n = 11), lymphocyte aggregation in fibrovascular cores (n = 10), mast cell infiltration (n = 8), and intralesional hemorrhage (n = 7). Uncommonly seen histological features included lymphoid cuff (n = 4), hemosiderin deposition (n = 5), foci of clear cell change (n = 4), intracytoplasmic vacuoles (n = 4), eosinophilic hobnail cells (n = 2), and infarct-type necrosis (n = 1). Two PRNRPs were concurrent with ipsilateral clear cell papillary RCC and clear cell RCC, respectively. By IHC, the tumors were consistently positive for GATA3, CK7, and PAX8. Fourteen out of 16 tumors showed a basolateral-membranous E-cadherin expression pattern, and 12/16 cases were positive for 34βE12.The expression of AMACR, CD10, and vimentin was either absent or only weak and focal. By targeted NGS, 13/14 evaluated PRNRPs harbored *KRAS* missense mutations involving c.35G>T resulting in p.G12V (7/13), c.35G>A resulting in p.G12D (4/13), and c.34G>T resulting in p.G12C (2/13). By FISH, 1/15 had gains of chromosomes 7 and 17, and 2/8 male cases had deletion of chromosomes Y. In conclusion, our study confirms that PRNRP is an indolent renal cell neoplasm with unique morphology, consistent immunohistochemical profile, and recurrent *KRAS* mutation. Our study expands the morphologic spectrum of PRNRP and provides further evidence supporting it as a novel entity.

## Introduction

Papillary renal cell carcinoma (PRCC), the second prevalent subtype among RCCs, has been divided into types 1 and 2 for more than two decades (1). Histologically, PRCC type 1 is characterized by papillary architecture covered by a single layer of cuboidal cells with scant pale or basophilic cytoplasm and low-grade nuclei under the criterion made by the World Health Organization/International Society of Urological Pathology (WHO/ISUP), whereas PRCC type 2 usually exhibits large pseudostratified cells with abundant eosinophilic cytoplasm and high WHO/ISUP nuclear grade (1–3). PRCC type 2 has worse prognosis than type 1 (2). The Cancer Genome Atlas (TCGA) research group revealed that PRCC type 1 was associated with *MET* mutations, while PRCC type 2 was a heterogeneous tumor at the molecular level involving *CDKN2A* silencing, *SETD2* mutations, and *TFE3* fusions (4). Genetic differences further explained the morphologic discrimination between these two types (4). In practice, however, it may be challenging to dichotomize PRCC as such, since well-sampled tumors frequently harbor mixtures of type 1 and 2 areas (5). Currently, the 2022 WHO classification eliminated the PRCC type 1/2 subcategorization, given the recognition of frequent mixed tumor phenotypes and the existence of entities with a different molecular background within the PRCC type 2 category (6). A subset of PRCCs that had granular eosinophilic cytoplasm but favorable prognosis has been designated as oncocytoid-type or oncocytic PRCC (7, 8). Subsequently, several studies that referred to varied inclusion criteria were performed and proposed various terminologies such as oncocytic PRCC with an inverted nuclear pattern and oncocytic low-grade variant of PRCC (9, 10). In 2016, WHO designated PRCC with voluminous granular eosinophilic cytoplasm and a monotonous layer of cells with low WHO/ISUP nuclear grade as oncocytic PRCC (3). However, emerging evidence suggests that oncocytic PRCC may not be an independent tumor entity, as oncocytic change can be noted in otherwise typical type 1 or 2 PRCC (11). In 2017, Saleeb et al. (12) subdivided PRCC into four types, of which PRCC type 4 showed morphology similar to that of oncocytic PRCC and was characterized by specific GATA3 immunoreactivity. In 2019, Al-Obaidy et al. (13) used the term “papillary renal neoplasm with reverse polarity” (PRNRP) for the first time and proposed that it should be distinguished from both PRCC types 1 and 2. Subsequently, the term “papillary renal neoplasm” not “papillary RCC” was widely adopted based on its extremely indolent behavior.

PRNRPs are composed of papillary or rarely tubular architectures with a single layer of uniform cuboidal cells with finely granular cytoplasm and apically located, low WHO/ISUP grade nuclei with inconspicuous nucleoli. Immunohistochemical staining for GATA3 and L1CAM along with the lack of vimentin expression is characteristic (13). The same group subsequently discovered that recurrent *KRAS* missense mutations at codon 12 of exon 2 may be a molecular hallmark for PRNRP, verifying the distinction from other renal cell neoplasms (14). More recently, several studies have also been published on this entity, reinforcing our understanding of its histologic and molecular genetic characteristics (15–19). In 2021, in its update on existing renal neoplasms, the Genitourinary Pathology Society (GUPS) has considered PRNRP to represent a distinct pattern/variant within the spectrum of PRCC (20). Most recently, type D papillary adenoma (PA) has been suggested to represent an analogue or a small-sized, clinically undetected PRNRP on the basis of their identical morphology, immunophenotype, and molecular genetics, broadening the concept of PRNRP (21–23).

In the current study, we identified 16 cases of PRNRP to further analyze the clinicopathological, immunohistochemical, and molecular features, expanding the morphologic spectrum of PRNRP and providing further evidence to support it as a novel entity.

## Materials and methods

### Case selection

Sixteen cases of PRNRP diagnosed between 2016 and 2021 from the files of three departments of pathology in China (The First Affiliated Hospital of Zhejiang University School of Medicine, Hangzhou; Ningbo Diagnostic Pathology Center, Ningbo; and Zhejiang Provincial People’s Hospital, Hangzhou) were retrieved. The clinical details and follow-up data were obtained from a review of the patients’ electronic records and from the physicians’ offices. For all cases, the hematoxylin–eosin (HE)-stained and immunohistochemical slides were reviewed and the diagnosis of PRNRP was further confirmed according to the diagnostic criteria proposed by Al-Obaidy et al. (13) in 2019. All tumors were graded according to the WHO/ISUP nuclear grading system (10) and staged on the basis of the eighth edition TNM staging system of renal neoplasms (24). This study was approved by the institutional ethics committee of Zhejiang Provincial People’s Hospital.

## Immunohistochemistry

All specimens were formalin-fixed and paraffin-embedded (FFPE). Tissues were sliced into 3-μm sections. Immunohistochemistry (IHC) was performed at a single laboratory (Zhejiang Provincial People’s Hospital, Hangzhou, China) using a Ventana Benchmark autostainer (Ventana Medical Systems, Tucson, USA). The following primary antibodies were used: PAX8 (Clone EP298, ZSGB-BIO, Beijing, China), GATA3 (Clone EP368, ZSGB-BIO, China), cytokeratin 7 (CK7, Clone EP16, ZSGB-BIO, China), 34βE12 (Clone 34βE12, ZSGB-BIO, China), E-cadherin (Clone EP6, ZSGB-BIO, China), alpha-methylacyl-CoA-racemase (AMACR, Clone 13H4, ZSGB-BIO, China), CD10 (Clone SP67, ZSGB-BIO, China), vimentin (Clone EP21, ZSGB-BIO, China), and CD117 (Clone YR145, Roche, China). The staining process was performed in accordance with the instructions and established positive and negative controls. We regarded the result as positive findings if the intensity was more than mild and evaluated the proportional score as follows: 0 negative, focal <50%; diffuse ≥50%.

### Fluorescence *in-situ* hybridization

Fluorescence *in-situ* hybridization (FISH) analysis was performed to identify the presence of chromosomal abnormalities including gains of 7 and 17, or losses of Y, as described previously (25, 26). The centromere-specific probe (CEP) 7, CEP17, CEP X, and CEP Y were all from Anbiping™ (Anbiping, Guangzhou, China). Only individual and well-delineated cells were scored. Overlapping cells were excluded from the analysis. Approximately 100 tumor cells were analyzed in the targeted region. Using established criteria, chromosomal gains were considered significant if present in greater than 20% of tumor cells (25), and chromosomal losses were considered significant if present in >45% of tumor cells (26). Gains or losses were considered artifactual if seen in less than 20% of cells and 45% of tumors, respectively.

### Targeted next-generation sequencing

For next-generation sequencing (NGS), 10 FFPE sections 5 µm thin containing >20% tumor cells confirmed by HE staining were used for genomic DNA and total RNA isolation. Genomic DNA and total RNA were extracted using a QIAamp Mini Kit (QIAGEN, Hilden, Germany). The DNA concentration was measured using a Qubit 4.0 Fluorometer (Thermo Fisher, Waltham, USA). A library was generated using RingCap™ loop-mediated amplification technology for the 13-gene panel (SpaceGen, Xiamen, China). This panel targeted the hotspot regions of *EGFR*, *KRAS*, *BRAF*, *PIK3CA*, *NRAS*, *HER2*, *MET*, *AKT1*, *KIT*, and *PDGFRA* with more than 500 hotspot mutations and 52 fusion variants of *ALK*, *ROS1*, and *RET* genes. Reads were generated on a MiniSeq platform (Illumina, San Diego, USA). Single-nucleotide variants (SNVs) and small insertions and deletions (InDels) with variant allele frequency more than 5% and gene fusions were annotated using a commercial mutation-reporting system (SpaceGen, Xiamen, China) and identified manually by Integrative Genomics Viewer.

## Results

### Clinicopathological characteristics

The clinicopathological data of the 16 cases were tabulated in **Table 1**. The patients included nine men and seven women, with age ranging from 47 to 80 years (median = 67.5 years, mean = 65 years). Most tumors (12/16) were incidentally identified by imaging studies while three presented with symptoms including back pain and hematuria; the remaining one (case 4, the smallest one) was incidentally identified in the radical nephrectomy specimen for end-stage renal disease (ESRD). Ten neoplasms affected the left kidney, and six the right. Except for case 4, all neoplasms were treated by partial nephrectomy. All tumors were confined to the kidney; hence, 15 were categorized as pT1 and 1 (case 3) was pT2, according to the eighth edition TNM staging system (24). With a median follow-up of 15 months (range, 1–62 months), no tumor recurrences, metastases, or disease-related deaths were identified for all the 16 patients.

**Table 1:** Clinicopathological characteristics of PRNRP.

Case no.	Sex/age (y)	Clinical manifestation	Laterality	Size (cm)	Stage	Follow-up (months)	Concurrent RCC	WHO/ISUP grade	Fibrous-capsule	Cystic change	Edematous/hyalinized papillae	Intracytoplasmic vacuoles	Clear cell change	Hobnail cells	Hemosiderin	Hemorrhage	Lymphocytes aggregation	Mast cells infiltration
1	F/76	Incidentally identified	Right	2.5	pT1a	NED (3)	N	1	N	Y, prominent	N	Y	Y	N	N	N	N	N
2	M/80	Incidentally identified	Right	2.8	pT1a	NED (3)	Y, CCPRCC	1	Y, lymphoid cuff	Y, prominent	Y	N	N	N	Y, extracellular	Y	N	N
3	M/67	Incidentally identified	Left	9.5	pT2a	NED (9)	N	2	Y, lymphoid cuff	Y, prominent	Y	N	N	N	N	N	Y	N
4	M/79	Incidentally identified	Right	0.4	pT1a	NED (1)	N	2	N	Y	N	Y	Y	Y	N	N	Y	N
5	M/54	Incidentally identified	Left	2.1	pT1a	NED (4)	N	2	Y	Y	Y	Y	Y	N	Y, extracellular	Y	Y	N
6	M/67	Back pain	Right	1.9	pT1a	NED (8)	N	1	N	Y	Y	Y	N	N	Y, intracellular	Y	Y	Y
7	M/55	Hematuria	Left	4.5	pT1b	NED (16)	N	2	N	Y, prominent	Y	N	Y	N	Y, intracellular	Y	Y	N
8	F/57	Incidentally identified	Right	1.2	pT1a	NED (39)	N	2	N	Y	Y	N	N	N	N	N	Y	Y
9	F/47	Incidentally identified	Left	2.6	pT1a	NED (62)	N	1	Y, lymphoid cuff	Y	Y	N	N	N	N	Y	Y	N
10	F/50	Incidentally identified	Right	1.0	pT1a	NED (15)	N	1	N	N	N	N	N	N	N	N	N	Y
11	F/76	Incidentally identified	Left	1.2	pT1a	NED (14)	N	1	N	Y	Y	N	N	N	N	N	Y	Y
12	M/70	Back pain, fever and hematuria	Left	6.0	pT1b	NED (7)	N	2	Y, lymphoid cuff	Y, prominent	Y	N	N	Y	N	Y, with infarct-type necrosis	N	Y
13	M/73	Incidentally identified	Right	1.0	pT1a	NED (42)	N	2	Y	N	N	N	N	N	N	N	N	N
14	M/70	Incidentally identified	Right	1.5	pT1a	NED (4)	N	2	N	Y	N	N	N	N	N	N	N	Y
15	F/68	Incidentally identified	Right	1.7	pT1a	NED (11)	Y, CCRCC	2	N	Y	Y	N	N	N	Y, intracellular	Y	Y	Y
16	F/51	Incidentally identified	Right	1.2	pT1a	NED (8)	N	2	Y	Y	Y	N	N	N	N	N	Y	Y

CCPRCC, clear cell papillary renal cell carcinoma; CCRCC, clear cell renal cell carcinoma; F, female; M, male; N, not; NED, no evidence of disease; WHO/ISUP, World Health Organization/International Society of Urological Pathology; Y, yes.

**Figure 1:**
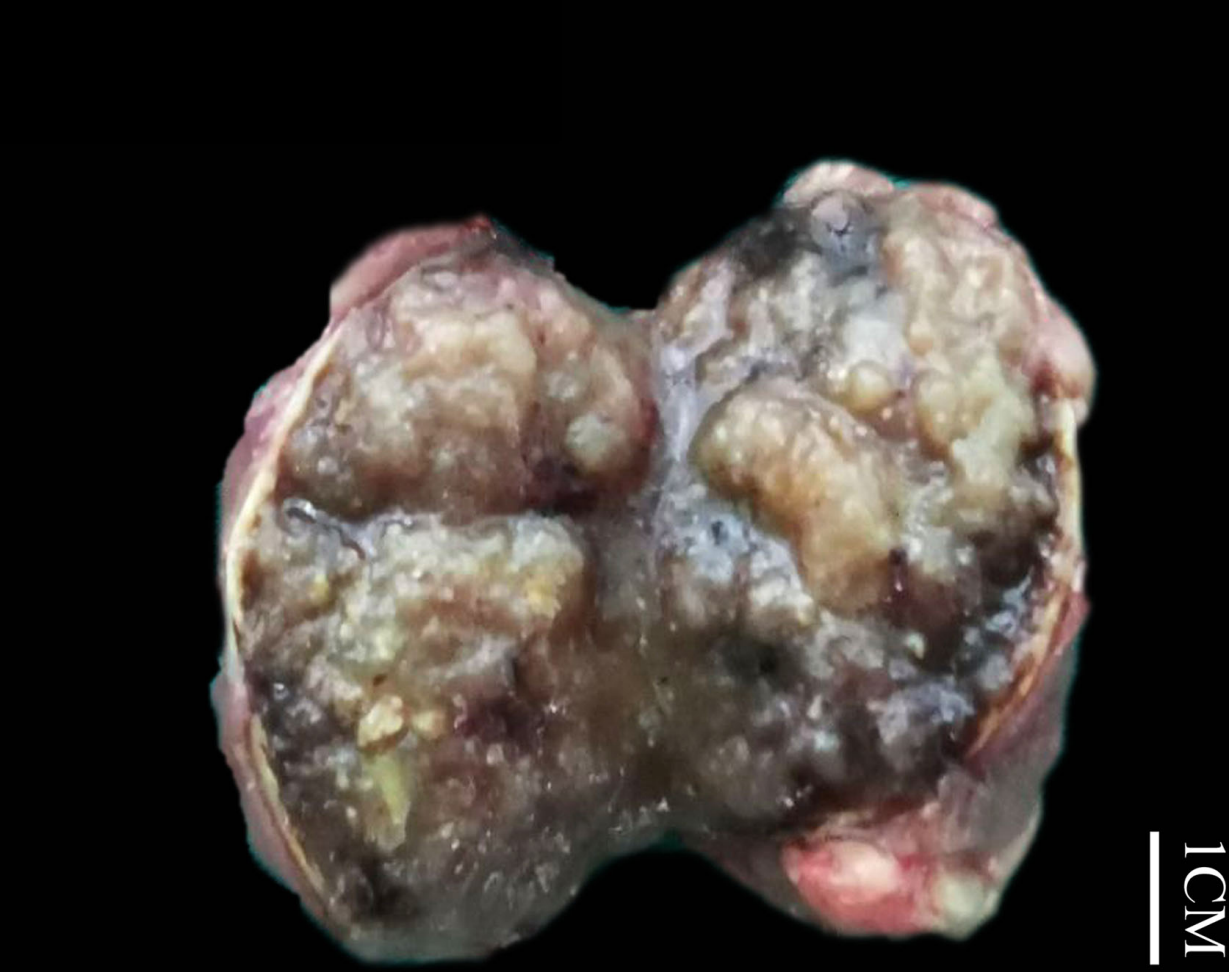
Gross examination showing an encapsulated and cystic PRNRP with a soft and friable, intracystic polypoid mass.

**Figure 2:**
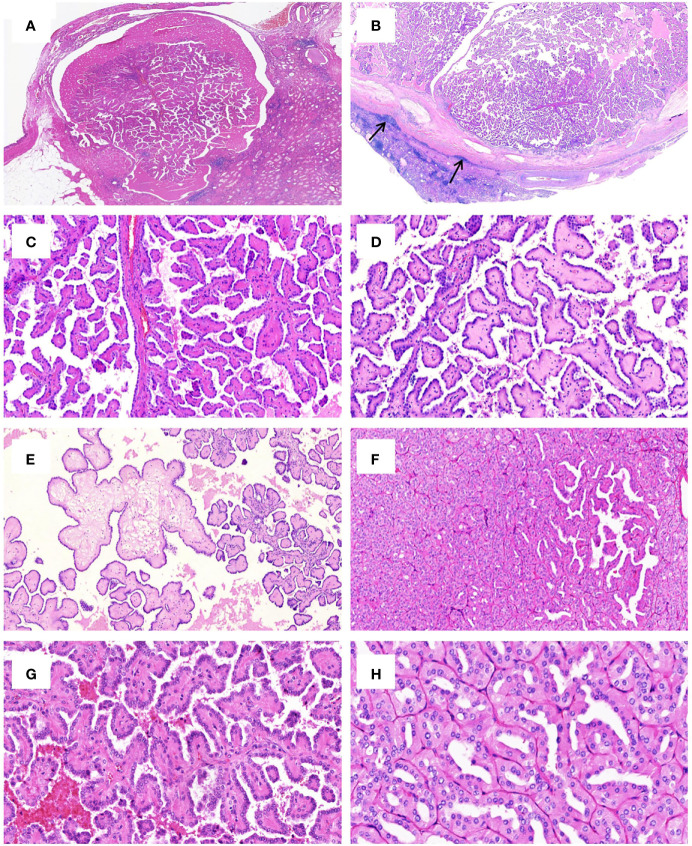
Histologic features of PRNRP. **(A)** Mixed solid and cystic tumor with eosinophilic proteinaceous material (HE, low magnification). **(B)** Thick fibrous capsule with peri-capsule lymphoid cuff (arrows, HE, low magnification). **(C)** Branching papillary architecture with delicate fibrovascular cores (HE, medium magnification). **(D)** Hyalinized papillae (HE, medium magnification). **(E)** Foci of edematous papillae (HE, medium magnification). **(F)** PRNRP consisting mostly of solid, densely packed tubules with only a minor papillary component (HE, medium magnification). The papillae (**G**, HE, high magnification) and tubules (**H**, HE, high magnification) are covered by oncocytic cells with inverted low-grade nuclei.

**Figure 3:**
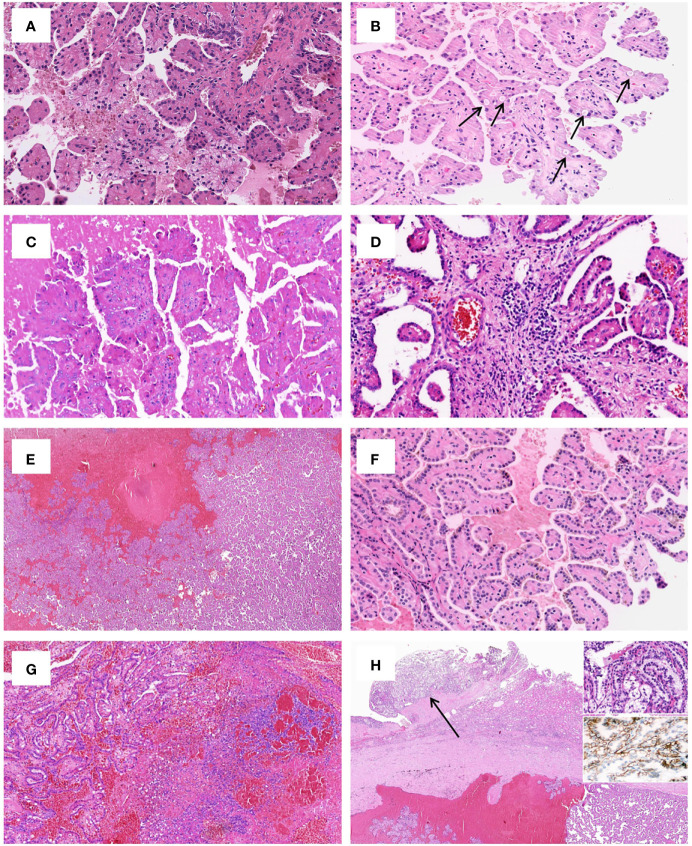
Uncommonly seen histologic features of PRNRP. **(A)** Clear cell change (HE, high magnification). **(B)** Intracytoplasmic vacuoles (arrows, HE, high magnification). **(C)** Eosinophilic hobnail cells (HE, high magnification). **(D)** Lymphocyte aggregation and scattered mast cell infiltration in the fibrovascular cores (HE, high magnification). **(E)** Cystic change with blood clots (HE, low magnification). **(F)** Intracellular hemosiderin deposition (HE, high magnification). **(G)** Infarct-type necrosis (HE, medium magnification). **(H)** PRNRP coexisting with an adjacent clear cell papillary renal cell carcinoma (arrow, HE, low magnification). ***Inserts*** showing clear cells with nuclei aligned circumferentially (right upper, HE, high magnification) and “cup-like” CAIX expression (right middle, high magnification).

Grossly, most tumors (13/16) were small tumors, less than 3 cm in size (median = 1.8 cm, mean = 2.6 cm; range, 0.4–9.5 cm). All tumors were well-demarcated or encapsulated and most (14/16) demonstrated at least a partially cystic appearance. Five cases with larger size were predominantly cystic, frequently with intracystic polypoid or papillary masses protruding into the cystic spaces (**Figure 1**); nine were predominantly solid with minor areas of cystic change; and two were completely solid. The tumors were typically soft and friable in texture and tan to light brown in color. Microscopically, at low power, the tumors were frequently mixed solid and cystic (**Figure 2A**). All tumors were circumscribed, and seven had a thick fibrous capsule, four of which had peri-capsule lymphoid cuff (**Figure 2B**). In all PRNRPs, except in one (case 13), the solid areas were composed predominantly of thin, branching papillary architecture (**Figures 2A–C**), with variable amounts of hyalinized or edematous papillae noted in 11 cases (**Figures 2D, E**); in case 13, the tumor consisted mostly of solid, densely packed tubules with only a minor papillary component (5%) (**Figure 2F**). The papillae and tubules were covered by a single layer of cuboidal cells with moderate, eosinophilic, or finely granular/oncocytic cytoplasm, indistinct cell membrane, and round, WHO/ISUP grade 1–2 nuclei typically located toward the apical surface away from the basement (**Figures 2G, H**). Foci of clear cell change and intracytoplasmic vacuoles were each observed in four cases (**Figures 3A, B**). In addition, eosinophilic hobnail cells were focally present in two cases (cases 4 and 12) (**Figure 3C**). Lymphocyte aggregation and scattered mast cell infiltration in the fibrovascular cores were notable in 10 and 8 PRNRP cases, respectively (**Figure 3D**). The cystic areas when noted were frequently filled with eosinophilic proteinaceous material or blood clots (**Figures 2A**, **3E**). Intralesional hemorrhage was identified in seven cases, and hemosiderin deposition was occasionally noted in five cases (intracellular in three and extracellular in two) (**Figure 3F**). In case 12, areas of infarct-type necrosis, due (putatively) to extensive intralesional hemorrhage, were identified (**Figure 3G**). Pseudostratification, psammoma bodies, foam cell clusters, coagulative-type tumor necrosis, or mitotic figures were absent in all tumors. All PRNRP cases were confined to the kidney, and none had microscopic lymphovascular invasion, perinephric fat invasion, or pelvicalyceal system involvement. A separate clear cell RCC (4.8 cm, WHO/ISUP grade 2) was observed in case 15, whereas a clear cell papillary RCC (1.4 cm) was present in case 2 (**Figure 3H**).

### Immunohistochemical results

**Figure 4:**
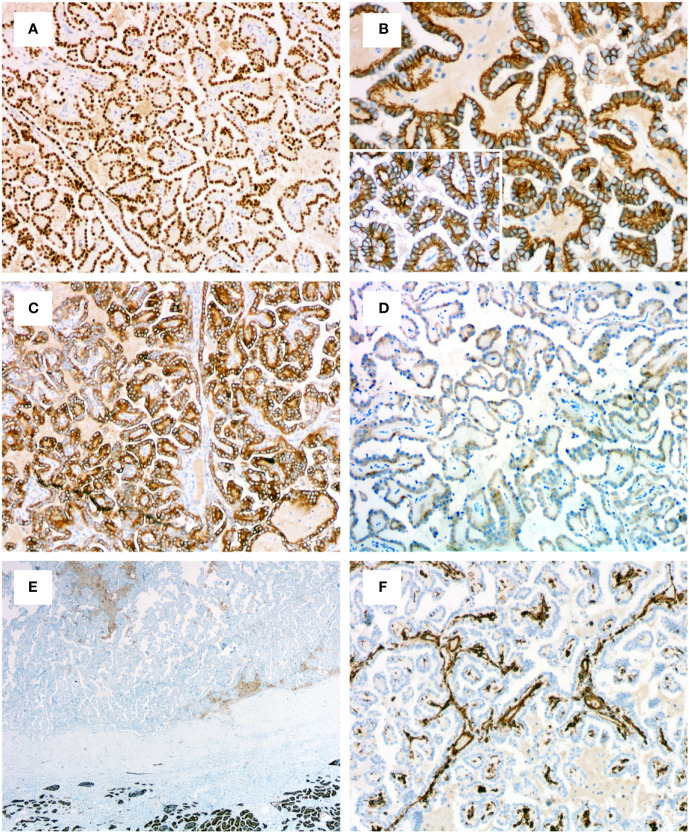
Immunohistochemical profiles of PRNRP. **(A)** Diffusely and strongly positive for GATA3 (medium magnification). **(B)** Basolateral-membranous/”cup-like” staining pattern of E-cadherin (high magnification). **(C)** Positive for 34βE12 (medium magnification). **(D)** Weakly positive for AMACR (medium magnification). Negative for CD10 (**E**, low magnification) and vimentin (**F**, medium magnification).

The immunohistochemical results for PRNRPs are summarized in **Table 2**. All PRNRPs showed strong and diffuse immunoreactivity to GATA3 (**Figure 4A**), CK7, and PAX8. Fourteen of the 16 tumors showed E-cadherin expression, with a diffuse, basolateral-membranous/”cup-like” staining pattern (**Figure 4B**), and 12/16 cases were strongly positive for 34βE12 (**Figure 4C**), with staining being diffusely in 11 and focally in 1. AMACR was weakly positive in 11 cases and negative in the remaining 5 cases (**Figure 4D**), and CD10 was focally and weakly positive in 3 cases and negative in the remaining 13 cases (**Figure 4E**). Vimentin was negative in 15 tumors and only focally positive in the remaining one (**Figure 4F**). All PRNRPs were completely negative for CAIX and CD117, while CD117 highlighted the mast cell infiltration in the fibrovascular cores of the papillae. The concurrent clear cell papillary RCC in case 2 showed a diffuse and strong expression of GATA3, 34βE12, CK7, and “cup-like” CAIX (**Figure 3H**).

**Table 2:** Immunohistochemistry staining features of PRNRP.

Case no.	GATA3	34βE12	AMACR	E-cadherin*	PAX8	CK7	CD10	CD117	Vimentin	CAIX
1	+	+	+ (weak)	+	+	+	–	–	–	–
2	+	+	+ (weak)	+	+	+	–	–	–	–
3	+	+	+ (weak)	+	+	+	–	–	–	–
4	+	+	–	–	+	+	–	–	–	–
5	+	+	+ (weak)	+	+	+	–	–	–	–
6	+	+	+ (weak)	+	+	+	–	–	–	–
7	+	+	–	+	+	+	–	–	–	–
8	+	+	–	–	+	+	+(focal and weak)	–	–	–
9	+	+	+ (weak)	+	+	+	–	–	+ (focal)	–
10	+	+	+ (weak)	+	+	+	–	–	–	–
11	+	+ (focal)	+ (weak)	+	+	+	+ (focal and weak)	–	–	–
12	+	–	–	+	+	+	–	–	–	–
13	+	–	+ (weak)	+	+	+	–	–	–	–
14	+	+	+ (weak)	+	+	+	+ (focal and weak)	–	–	–
15	+	–	+ (weak)	+	+	+	–	–	–	–
16	+	–	–	+	+	+	–	–	–	–

*basolateral-membranous staining pattern.

### Targeted NGS and FISH findings

**Figure 5:**
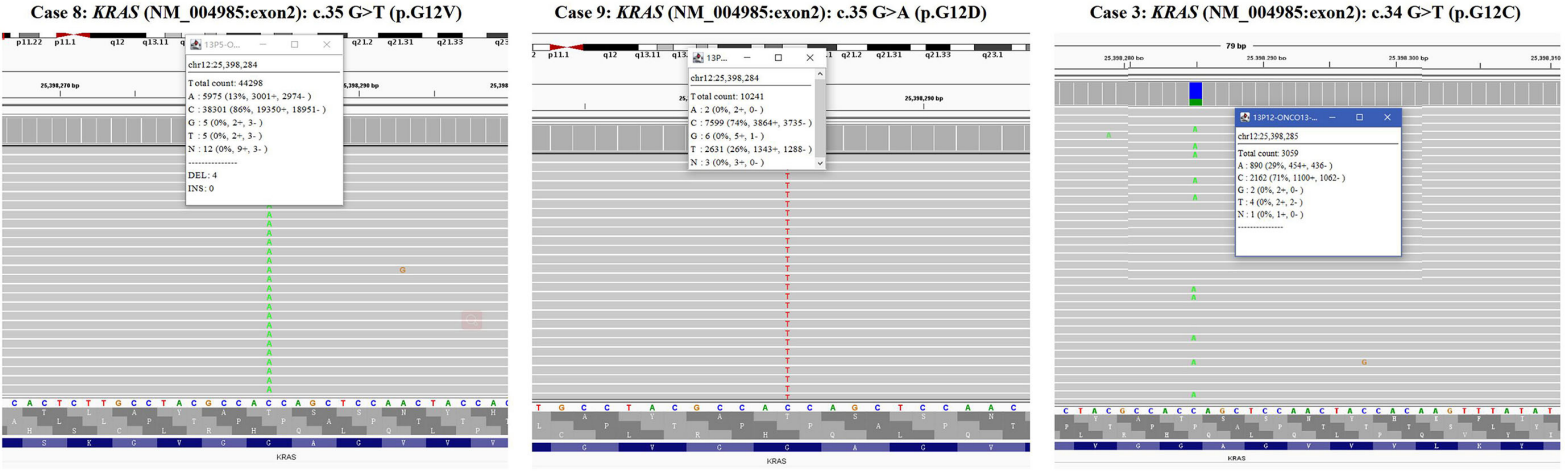
*KRAS* mutations in PRNRP. Integrative Genomics Viewer screenshot of the representative *KRAS* mutation hotspot, including c.35 G>T (p.G12V) (case 8), c.35G>A (p.G12D)(case 9), and c.34G>T(p.G12C)(case 3).

**Figure 6:**
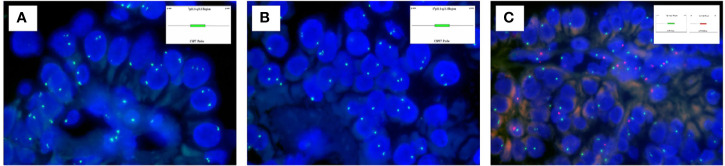
Fluorescence *in-situ* hybridization findings of PRNRP. **(A)** Trisomy 7 (case 1). ***Inset*** indicating the schematic diagram of the centromere-specific probe of chromosome 7. **(B)** Trisomy 17 (case 1). ***Inset*** indicating the schematic diagram of the centromere-specific probe of chromosome 7. **(C)** Deletion of chromosome Y (case 13). ***Insets*** indicating schematic diagrams of the centromere-specific probe of chromosome X (green) and chromosome Y(red).

The targeted NGS and FISH findings are summarized in **Tables 3** and **4**. The targeted NGS was performed in 14 out of 16 PRNRP cases. The targeted NGS was not performed in the remaining two cases, because their quality was not suitable for targeted sequencing. PRNRP tumors exhibited *KRAS* missense mutations in 13 out of the 14 cases (93%) by targeted NGS. These mutations were due to a c.35 G>T (7/13, 54%), c.35G>A (4/13, 31%), and c.34G>T(2/13, 15%) substitution, resulting in p.G12V, p.G12D, and p.G12C alterations, respectively (**Figure 5**). The allele frequency (AF) ranged from 9.2% to 33%. No mutations of other genes in the panel were identified in any of the PRNRPs. No fusion genes were detected. No *KRAS* mutations were found in either concurrent clear cell RCC or clear cell papillary RCC. By FISH analysis, one of the 15 PRNRP cases analyzed (case 1) demonstrated trisomy 7 and 17 (**Figures 6A, B**). Chromosome Y deletion was present in two of eight male cases examined (case 13 and 14)(**Figure 6C**).

**Table 3:** Targeted next-generation sequencing findings of PRNRP.

Case no.	SNV	Amino acid changes	AF (%)
1	c.35G>T	p.G12V	32.61%
2	c.35G>T	p.G12V	32.98%
3	c.34G>T	p.G12C	29.66%
4	ND	——	——
5	ND	——	——
6	c.35G>T	p.G12V	9.17%
7	c.35G>A	p.G12D	14.28%
8	c.35G>T	p.G12V	13.29%
9	c.35G>A	p.G12D	25.66%
10	c.34G>T	p.G12C	30.97%
11	c.35G>A	p.G12D	18.14%
12	c.35G>A	p.G12D	10.31%
13	c.35G>T	p.G12V	9.72%
14	Negative	Negative	Negative
15	c.35G>T	p.G12V	20.39%
16	c.35G>T	p.G12V	20.63%

AF, allele frequency; ND, not done; SNV, single-nucleotide variant.

**Table 4:** Fluorescence *in-situ* hybridization findings of PRNRP.

	Chromosome 7			Chromosome 17			Chromosome Y	
Case no.	1G(%)	2G(%)	3G(%)	1G(%)	2G(%)	3G(%)	1G(%)	1R1G(%)
1	6	71	23	4	76	20	——	——
2	4	91	5	4	92	4	5	95
3	15	83	2	14	84	2	2	98
4	ND	ND	ND	ND	ND	ND	ND	ND
5	22	75	3	39	58	3	3	99
6	28	66	6	35	63	2	2	98
7	21	67	2	31	63	5	1	99
8	24	76	0	31	65	4	——	——
9	18	77	5	21	78	1	——	——
10	5	92	3	68	21	11	——	——
11	10	87	3	40	58	2	——	——
12	33	64	3	31	66	3	25	75
13	5	92	3	55	34	11	68	32
14	41	56	3	49	47	4	76	24
15	45	49	6	39	54	7	——	——
16	7	89	4	37	56	7	——	——

G, green; ND, not done; R, red.

## Discussion

RCC featuring papillary architecture and eosinophilic or oncocytic cytoplasm represents a heterogeneous disease, encompassing an increasing number of tumor variants, such as PRNRP, PRCC type 2, MiT family translocation RCC, biphasic hyalinizing psammomatous RCC, acquired cystic disease-associated RCC, and fumarate hydratase (FH)-deficient RCC. PRNRP was firstly designated as its current name in 2019 by Al-Obaidy and colleagues (13). It accounts for 1.3% to 9.1% of all PRCCs according to the previous reports (13, 17, 18, 22, 27), and to date there are a total of approximately 160 cases of PRNRP that have been published in the English-language literature. In the current study, we were able to identify 16 cases of PRNRP from three tertiary medical institutions between 5 years during which more than 350 PRCCs have been diagnosed, further indicating the rarity of this tumor type.

In agreement with previous studies, the 16 PRNRPs in our cohort presented mostly with an incidentally identified mass during the imaging study and showed a slight male predilection, and had small tumor size, low TNM stage, low WHO/ISUP nuclear grade, and a favorable prognosis at follow-up, without any recurrence, metastasis, or tumor-related death after surgical excision (13–19, 22, 23). Although most are solitary tumors, previous studies have demonstrated that a subset of PRNRPs may have multiple tumors as defined by presence of ≥2 unilateral or bilateral tumors, particularly for those with small size which are usually clinically undetectable (13, 22, 23). A recently published paper by Wei et al. (19) comprehensively reviewed the 100 reported cases of PRNRP and emphasized that this tumor was frequently a cystic tumor and at least a partially cystic change can be noted in 62% cases. Additionally, Al-Obaidy et al. (23) documented cystic expansion with intracystic papillary proliferation in 7 of their 16 clinically detected (≥5 mm) neoplasms. In line with these results, in our cohort, 14 cases displayed at least a partially cystic appearance and 5 larger tumors were predominantly cystic, frequently with intracystic polypoid or papillary masses protruding into the cystic spaces. Chang et al. (22) demonstrated that PRNRP showed a significantly higher association with ESRD compared with PRCC type 2. Similarly, Al-Obaidy et al. (23) showed that 12 of 35 clinically undetected (<5 mm) neoplasms were discovered on nephrectomy specimens performed for ESRD. In the current study, the smallest one (case 4, 0.4 cm) was clinically undetected and was incidentally identified in the nephrectomy specimen for ESRD. Concurrent ipsilateral renal tumor is not uncommon in PRNRP, and Lee et al. (28) reported a PRNRP with a *KRAS* mutation and a co-occurring clear cell RCC with a *PIK3CA* mutation in 2020. Most recently, Al-Obaidy and colleagues (23) found that 26 of 50 PRNRPs had other concurrent tumors of different histologic subtypes in the ipsilateral kidney, particularly for those with diameters less than 5 mm. In their study, the concurrent renal tumors included PRCCs, clear cell RCCs, acquired cystic disease-associated RCCs, chromophobe RCCs, and oncocytomas. In our cases, two PRNRPs coexisted with clear cell RCC and clear cell papillary RCC, respectively. To our knowledge, the latter tumor type was the first time to be reported to co-occur with PRNRP.

Histologically, PRNRP was originally documented by Al-Obaidy and colleagues (13) as a well-demarcated or encapsulated neoplasm displaying delicate and thin, arborizing papillary patterns or predominantly solid tubular growth in occasional cases. In that study, a minority of tumors showed thicker and hyalinized papillary cores or edematous papillae with cystically dilated tips filled with clear to eosinophilic fluid containing floating foamy macrophages. However, subsequent multiple studies reported that hyalinized or edematous papillae could be observed in the majority of PRNRP cases (26/30 in Kim et al. (15), 7/10 cases in Tong et al. (16), 9/14 in Kiyozawa et al. (18), and 11/16 in our cohort). The lining epithelium typically consisted of a monolayer of cuboidal to columnar cells with moderate to abundant, finely granular eosinophilic cytoplasm frequently with intracytoplasmic clear vacuoles or lumens, and apically located round, bland-appearing nuclei with inconspicuous nucleoli. Rare areas of nuclear clearing, wrinkled nuclear contours, and mild nuclear enlargement were also observed. Al-Obaidy et al. (13) did not identify psammoma bodies, intracellular hemosiderin, tumor necrosis, tight clusters of foamy macrophages, and mitoses in all tumors. Subsequent studies expanded the morphological spectrum of PRNRP to include eosinophilic hobnail cells (16, 22, 23), clear cell change on the tumor cells (18, 22, 23), peritumoral lymphoid cuff (15, 22), foamy histiocyte aggregation (15, 16, 18, 22), intracellular hemosiderin (22, 23), and lymphocyte or mast cell infiltration in the stroma (15, 16, 22, 23). These morphologies are typically focal and only present in a small subset of cases. The cases in our cohort had comparable findings except for foamy histiocyte aggregation, which were not identified. Tong et al. (16) reported that 8/10 cases had focal areas showing hobnail cells with abundant eosinophilic cytoplasm. Chang et al. (22) demonstrated that 60% (6/10) of their cases had focal hobnail features. In the most recently published to date study on the largest series of PRNRPs, Al-Obaidy et al. (23) found that hobnail conformation was present in only 3 out of the 50 cases. We found eosinophilic hobnail features in 2 of the 16 cases. Despite the presence of hobnail features, all of these reported cases had non-overlapping and WHO/ISUP low-grade nuclei. Although Kim et al. (15) reported 4/30 (13%) of their cases with WHO/ISUP grade 3 nuclei, our cases and other studies only found WHO/ISUP grade 1 or 2 nuclei in PRNRPs. Foamy histiocytes are uncommonly seen in PRNRPs; when present, they typically appeared as loose, scattered macrophages floating within the edematous cores (13), contrasting sharply to the tight clusters in papillary RCC. We identified foci of infarct-type necrosis in our case 12, which is exceptional for PRNRP, as tumor necrosis was consistently absent in previously documented cases. We speculate that infarct formation in this case is related to intralesional hemorrhage and cystic degeneration, which leads to the increased pressure in the cystic cavity, further resulting in ischemic infarction of the tumor. However, it should be mentioned that the cysts are commonly filled with proteinaceous fluid or blood clots and may contain floating degenerated cell debris with hemosiderin deposition, which may be confused with necrosis; however, true tumor type coagulative necrosis is never present in PRNRPs (23).

In the current study, all PRNRP cases exhibited a diffuse and strong expression of PAX8, CK7, and GATA3, whereas the expression of AMACR, CD10, and vimentin was either absent or only weak and focal, and the tumor cells were completely negative for CD117 and CAIX. The immunohistochemical profiles of PRNRP in our cohort are in line with the results in a recent meta-analysis by Wei and colleagues (19). In three previous reports, it was disclosed that PRNRP usually displayed a diffuse L1CAM expression typically in a basolateral and lateral membrane pattern, leading to a “cup-like” staining appearance (13, 15, 23). As L1CAM is not available in our laboratory, we performed immunostaining for E-cadherin and found that it was expressed in 14/16 (87.5%) of our PRNRP cases and all the 14 cases showed a “cup-like” staining pattern identical to that of L1CAM. Only one previous study has investigated the expression of E-cadherin in PRNRP. Kim et al. (15) found that 23/30 (77%) of their cases showed positive reactivity to E-cadherin with the expression being significantly higher than both PRCC types 1 and 2; however, they did not specify the staining pattern in their study. In the present study, we found that 12/16 (75%) PRNRPs were strongly positive for 34βE12, with staining being diffusely in 11 and focally in 1, consistent with the results reported by Zhou et al. (17), who found that all the seven cases of PRNRP in their study strongly expressed 34βE12. These data suggest that 34βE12 may serve as a sensitive marker for the diagnosis of PRNRP. Co-expression of GATA3 and 34βE12 is relatively rare in renal cell tumors and is often seen in tumors of distal nephron or collecting duct origin, such as collecting duct carcinoma (29, 30) and clear cell papillary RCC (31, 32). For the latter, studies have demonstrated that both GATA3 and 34βE12 can be used as sensitive and specific markers for its diagnosis and differential diagnosis (31, 32). With regard to the cell origin, the co-expression of GATA3 and 34βE12 in PRNRP may also point to its distal nephron origin. Using public data sets, Tong et al. (16) found that PRNRP shared similar gene expression profiles with cortical collecting duct, suggesting that PRNRP may potentially originate from the distal renal tubule. In addition, a negative or focal/weak expression of proximal renal tubule markers, such as vimentin, CD10, CD15, and AMACR (33), also supports this speculation. In a most recently published abstract, using unsupervised clustering analysis, Park and colleagues (34) found that PRNRPs formed a tight group on tSNE and were distant from PRCCs while close to clear cell papillary RCCs, further supporting this hypothesis.

At the molecular genetic level, NGS revealed in our cohort that PRNRP contained *KRAS* mutation at a high frequency (13/14, 93%). The one without *KRAS* mutation (case 14) showed typical morphological and immunohistochemical features consistent with PRNRP. *KRAS* missense mutations clustered in codon 12 in exon 2, with c.35G>T (p.G12V, 54%), c.35G>A (p.G12D, 31%), and c.34G>T (p.G12C, 15%). The AF ranged from 9.2% to 33%, supporting the somatic origin. There was no correlation between histologic phenotype and *KRAS* mutant genotype. Case 13 showed a predominantly tubular growth pattern which had immunohistochemical features identical to those with prominent papillary morphology. This case harbored a *KRAS* mutation with c.35G>T (p.G12V). *KRAS* mutation was identified in 57%–93% of PRNRPs in previous studies (14–16, 18, 19, 22, 23), with an overall frequency of 85% and the most common *KRAS* mutation being p.G12V (54%), as documented by Wei et al. (19). One *KRAS*-mutated PRNRP was reported to harbor a G12A/V/D complex mutation (23). In the past research, other somatic mutations detected by NGS in PRNRP included mutations in *BRCA2*, *BRIP1*, *RAD50*, *TP53*, and *BRAF* (14, 15, 18). Chang et al. (22) demonstrated recurrent activating *KRAS* mutation in six of eight cases of type D PA, which shows identical morphology and immunophenotype to PRNRP (21). Additionally, Al-Obaidy et al. (23) found *KRAS* mutation in 44% (15/34) of the microscopic (<5 mm) PRNRPs, and in 88% (14/16) of the clinically detected (≥5 mm) ones. These findings indicate that *KRAS* mutation may be an early molecular event in the tumorigenesis or progression of PRNRP, and type D PA may represent an analogue or a small-sized PRNRP (22, 23). *KRAS* missense mutation rarely appeared in other types of RCC, and only Raspollini et al. (35) reported a case of clear cell RCC harboring *KRAS* mutation. Several previous studies have documented that *KRAS* mutation was absent in clear cell RCC, PRCC (both types 1 and 2), chromophobe RCC, and clear cell papillary RCC (14, 15, 23). According to TCGA database, mutation of *KRAS* occurred in 0.7% (2/279) of PRCCs and 0.2% (1/451) of clear cell RCCs (14–16). Five tumors harboring a *KRAS* mutation have been registered as PRCC in the TCGA database; however, different groups have reviewed these tumors independently and concluded that three of these tumors in fact represented PRNRP (14–16). These above findings suggest that *KRAS* mutation is a consistent and unique finding in PRNRP and can serve as a powerful molecular tool for the accurate diagnosis of PRNRP when at challenging settings. Bioinformatics analysis has shown that a prominent *KRAS* signature is associated with activation of several important transcription factor networks, including GATA3 (36). In addition, overexpression of GATA3 was found in *KRAS*-driven lung cancer cells and further promoted the oncogenesis *via* microRNAs (37). These aforementioned evidence may explain the potential link between the genotype and immunophenotype of PRNRP; however, the function of mutated *KRAS* in the pathogenesis or progression of PRNRP requires further investigations. Trisomy of chromosome 7 and/or 17 and/or deletion of Y chromosome have been shown to be chromosomal abnormalities characteristic of PRCC (38). Prior studies using FISH analysis demonstrated that a subset of PRNRPs shared these specific chromosomal abnormalities with PRCCs (13, 17). However, Kiyozawa et al. (18) and Wei et al. (19) found that no cases of PRNRP had gains of chromosome 7 and/or 17, or loss of the Y chromosome, using copy number alteration analysis and chromosomal microarray analysis, respectively. In the present study, FISH study revealed trisomy 7 and 17 in only one of the 15 PRNRP cases analyzed. Chromosome Y deletion was identified in two of the eight male cases examined. These above findings suggest that the presence of trisomy 7 and/or 17 and loss of Y chromosome in PRNRP is likely to represent a random rather than a recurrent event, contrasting to those in PRCC.

A potential limitation of our study is the relatively short follow-up time for these tumors, although the vast majority of tumors were in the pT1 stage with 1 in the pT2 stage. The current data seem to be inadequate to make a conclusion about the long-term outcomes of this emerging neoplasm entity. Because PRNRP is overall a low-stage tumor with an indolent biological behavior, it is important to differentiate PRNRP from other renal tumors featuring papillary architecture and oncocytic cytoplasm. Differentiation of PRNRP from PRCC type 2 is sometimes difficult, as both of them may display similar histologic appearances. Although reverse polarity of nuclei is one of the characteristic features of PRNRP, it can be focally observed in a subset cases of PRCC type 2 (15). In addition, pseudostratification, a commonly seen feature in PRCC type 2, has also been reported in a few PRNRPs (13, 22). In cases with overlapping histology, immunohistochemical staining and *KRAS* mutation analysis can help for making a correct diagnosis. Positivity for GATA3 and 34βE12 along with negativity for CD10 and vimentin could be useful for supporting PRNRP, while detection of *KRAS* mutation by molecular genetics can further confirm the diagnosis. Since a few RCCs with MiT family alterations may demonstrate oncocytic and papillary RCC-like morphology with reverse polarity of nuclei, it is necessary to exclude these tumors by FISH assays (39). As mentioned above, a diffuse and strong expression of both GATA3 and 34βE12 can also be noted in clear cell papillary RCC (31, 32), which may be confused with PRNRP with focal clear cell change. However, clear cell papillary RCC is characterized by a papillary growth of low-grade clear cells with circumferentially aligned nuclei and lack of the oncocytic cytoplasm and inverted nuclei characteristic of PRNRP. FH-deficient RCC is a highly aggressive renal cancer and shows quite variable morphology; it can have prominent papillary architecture and oncocytic cytoplasm mimicking PRNRP. However, FH-deficient RCC usually has nuclear features of large reddish inclusion-like nucleoli surrounded by a clear halo that can suggest the diagnosis. Loss of FH and 2 succinyl cysteine positivity by IHC and/or detection of *FH* mutation (either germline or somatic) can further confirm the diagnosis (20, 40). Lastly, PRNRP with a prominent tubular pattern may be confused with recently characterized low-grade oncocytic tumor or eosinophilic vacuolated tumor; however, these tumors are frequently associated with mutations involving TSC/MTOR pathways and typically lack the inverted nuclei and GATA3 positivity in PRNRP (41–43).

## Conclusions

In summary, PRNRP is a rare renal tumor with an indolent clinical course. We confirm the previous reports that PRNRP is pathologically characterized by papillary or tubulopapillary architecture with frequently cystic change, low-grade tumor cells with oncocytic cytoplasm and inverted nuclear location, diffuse and strong expression of GATA3 and 34βE12, and recurrent *KRAS* mutation. We further expand the histologic spectrum of PRNRP to include the presence of infarct-type necrosis, concurrent with clear cell papillary PRCC, and basolateral and lateral membrane expression of E-cadherin by IHC. Our study further supports that PRNRP should be considered as a novel renal cell tumor entity.

## Data availability statement

The original contributions presented in the study are included in the article/supplementary material. Further inquiries can be directed to the corresponding author.

## Ethics statement

The studies involving human participants were reviewed and approved by the Institutional Review Board Committee of Zhejiang Provincial People’s Hospital, Affiliated People’s Hospital, Hangzhou Medical College. Written informed consent for participation was not required for this study in accordance with the institutional requirements.

## Author contributions

MS, XY, and MZ conceptualized the study concept and design. MS, YB, and HZ provided the follow-up information and analyzed the clinical data. MS, XY, YB, HZ, XH, XT, and MZ were in charge of the histologic diagnosis and interpretation of immunohistochemistry and analysis of the results of the targeted next-generation sequencing. MS and XY drafted the manuscript. GR, GC, and MZ revised the manuscript. All authors contributed to the article and approved the submitted version.

## Funding

This work was supported by Zhejiang Provincial Natural Science Foundation (LY21H160052) and Zhejiang Provincial Medicine and Health Research Foundation (2019KY020, 2019KY029, and WKJ-ZJ-1929). The funders did not have any role in the design and conduct of the study, the analysis and interpretation of the data, and preparation of the manuscript.

## Conflict of interest

The authors declare that the research was conducted in the absence of any commercial or financial relationships that could be construed as a potential conflict of interest.

## Publisher’s note

All claims expressed in this article are solely those of the authors and do not necessarily represent those of their affiliated organizations, or those of the publisher, the editors and the reviewers. Any product that may be evaluated in this article, or claim that may be made by its manufacturer, is not guaranteed or endorsed by the publisher.
